# Supplementary figures and images for: The establishment of variant surface glycoprotein monoallelic expression revealed by single-cell RNA-seq of Trypanosoma brucei in the tsetse fly salivary glands
Source: PLoS Pathog. 2021 Sep 20;17(9):e1009904. doi: 10.1371/journal.ppat.1009904 (PMC8509897; doi:10.1371/journal.ppat.1009904)

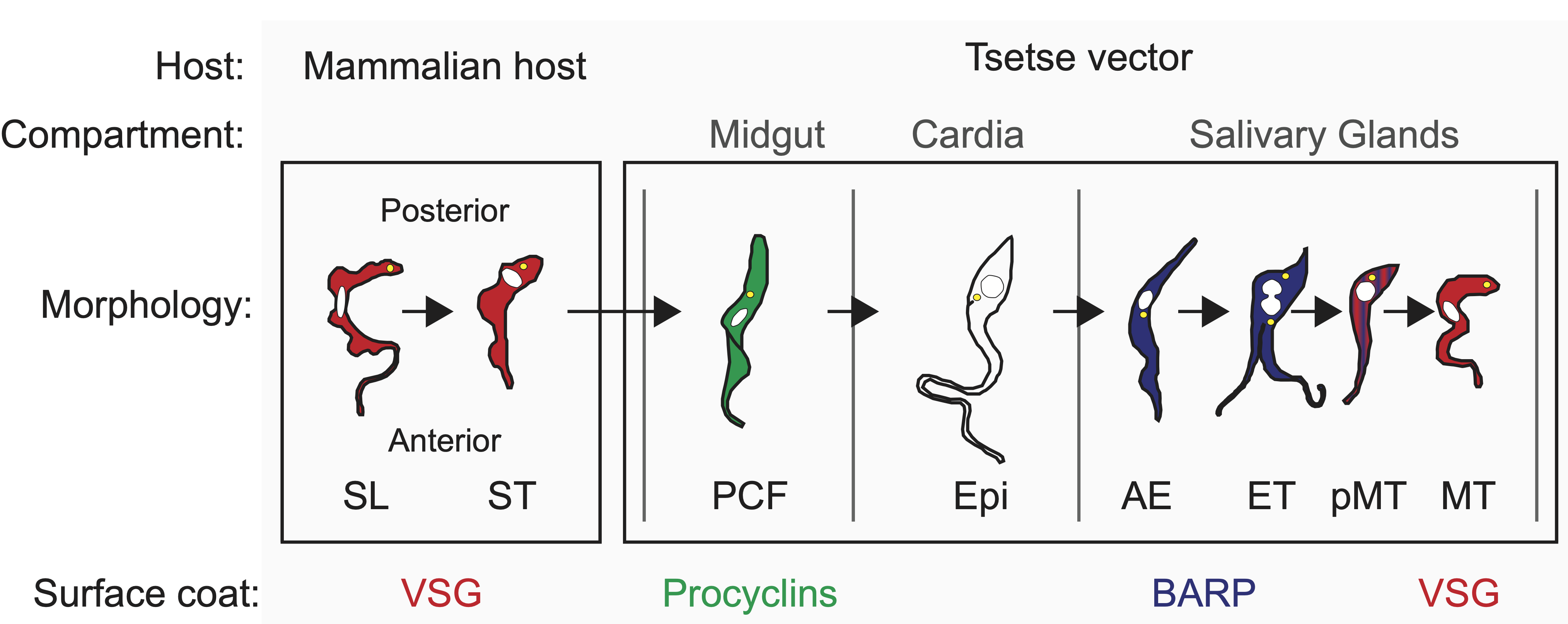

Supplement: S1 Fig — The morphology and surface protein expression of trypanosomes during differentiation is depicted in chronological order from left to right. Cell types shown here are SL: slender bloodstream form; ST: stumpy; PCF: procyclic form; Epi: epimastigote; AE: attached epimastigote; ET: epimastigote-trypomastigote dividing cell; pMT: pre-metacyclic cell; MT: metacyclic. The nucleus is depicted as a white circle / oval and the kinetoplast (mitochondrial DNA) as a yellow circle. In trypomastigote cells, the kinetoplast is posterior to the nucleus, e.g. in PCF. In epimastigote cells, the kinetoplast is anterior to the nucleus, e.g. in Epi. Cells are coloured according to the family of surface protein expressed. (TIF) [file ppat.1009904.s001.tif]

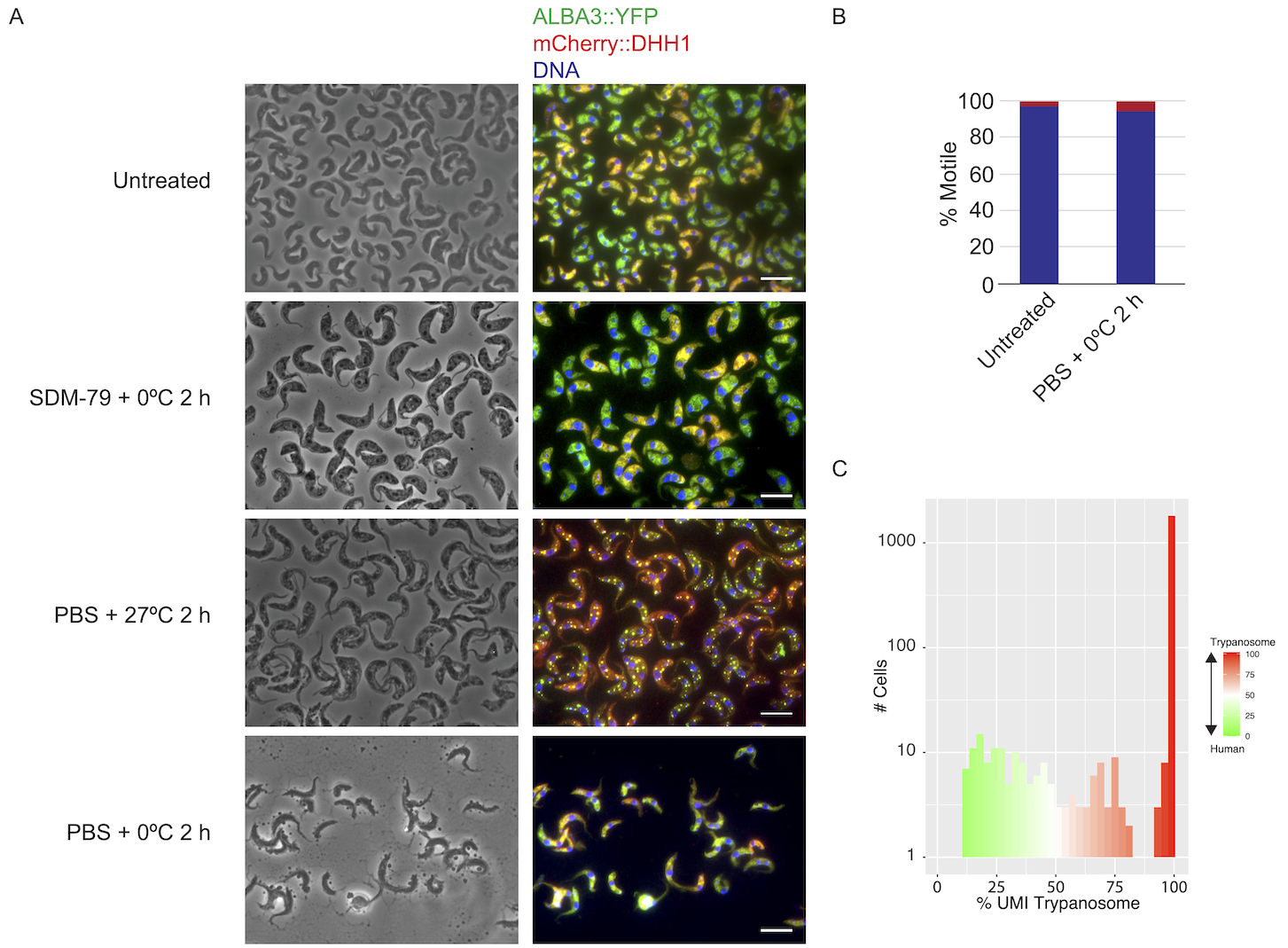

Supplement: S2 Fig — A. Trypanosomes collected by centrifugation and resuspended in either SDM-79 medium, or PBS and incubated at either 0°C or 27°C. Scale bar 10 μm. B. Results of live/dead assay based on trypanosome motility, by scoring live cells subjected to phase contrast microscopy. We assessed the ability of the parasites to survive starvation at 0°C for 2h in PBS. C. Histogram shows the percentage of stringently aligned reads (mapQ > 40) to the trypanosome or human transcriptomes. (TIF) [file ppat.1009904.s002.tif]

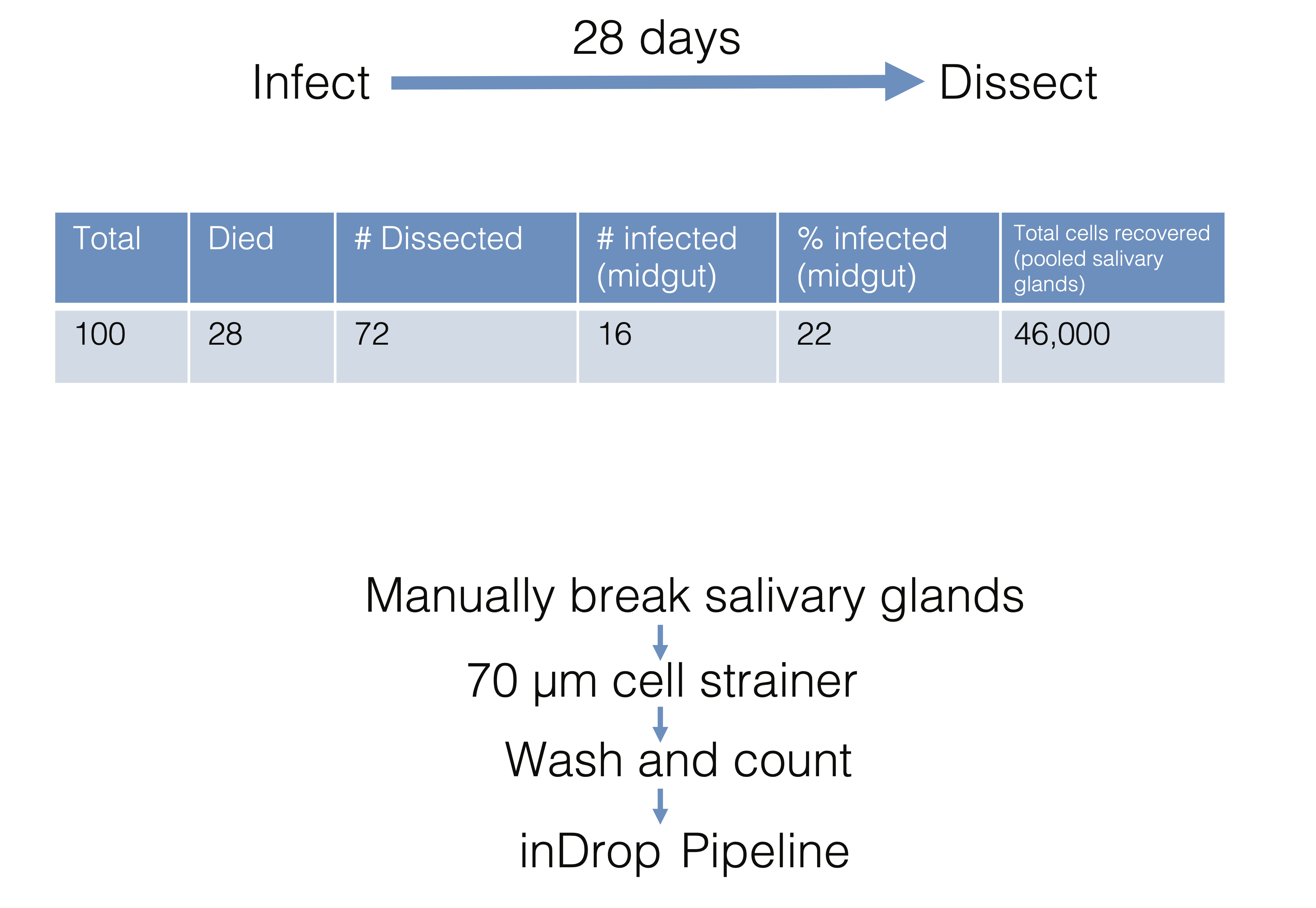

Supplement: S3 Fig — Flies were infected for 28 days. The table shows the characteristics of the dissection. Below is the workflow for dissections and sample collection. (TIF) [file ppat.1009904.s003.tif]

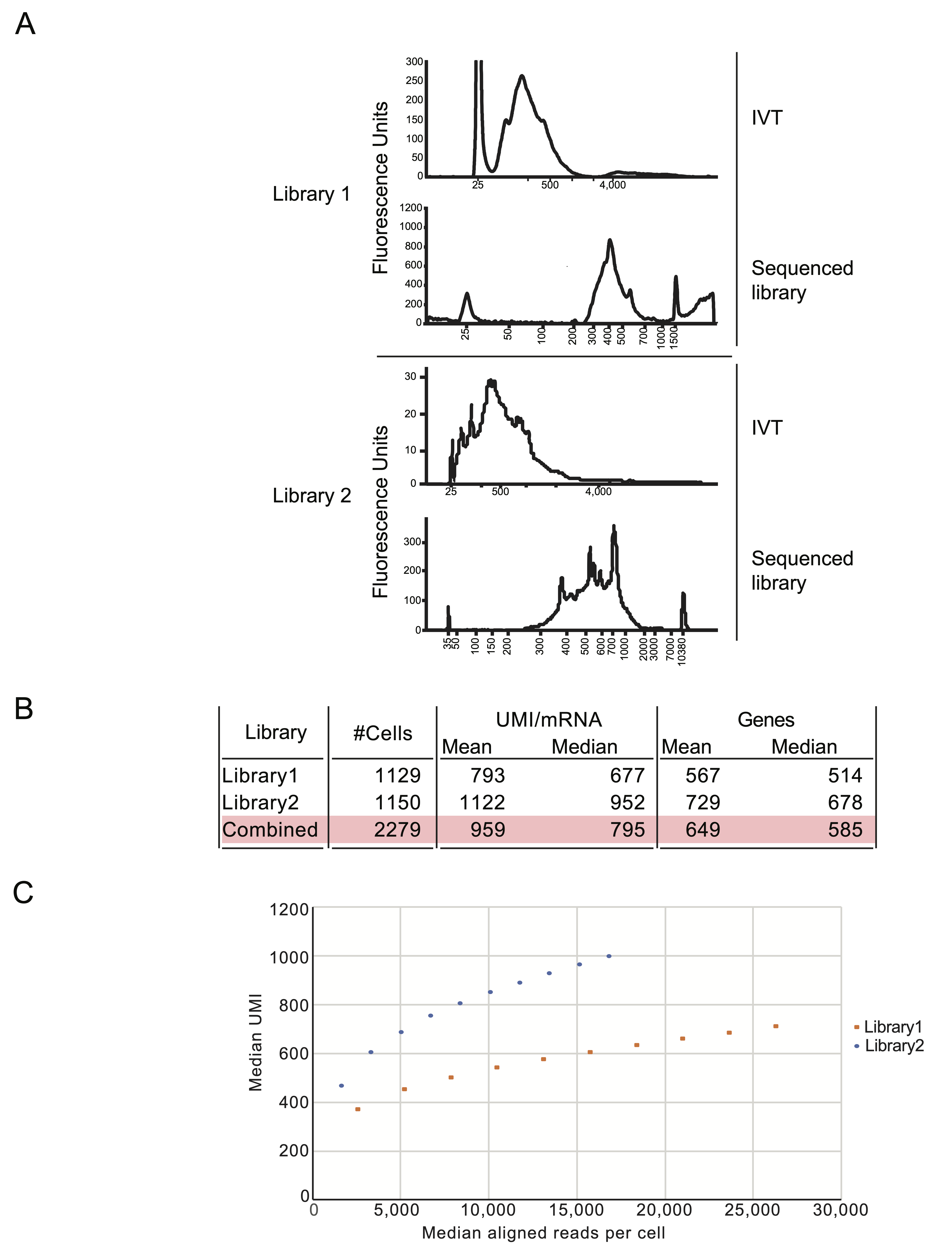

Supplement: S4 Fig — A. Tapestation or Bioanalyzer traces for IVT and library PCR reactions to construct libraries sequenced in this study. B. Table shows the number of cells recovered, and key parameters including UMIs and genes captured. C. Subsetting analysis of the data. Reads were subsampled using samtools [79] in 10% intervals from 10% to 100% before being re-counted using UMI-tools [81]. Aligned reads per cell were counted using a custom script (10.5281/zenodo.3974628). (TIF) [file ppat.1009904.s004.tif]

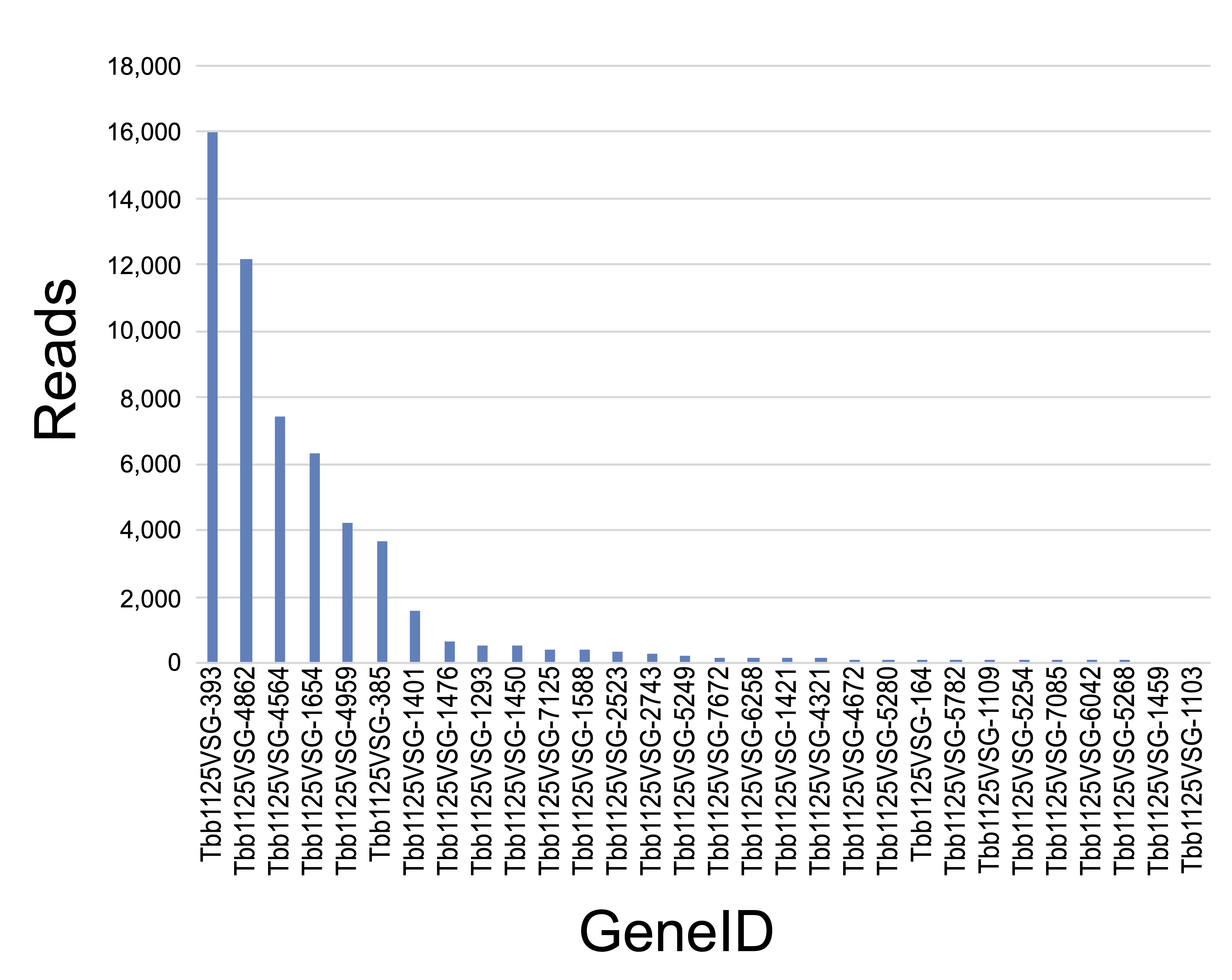

Supplement: S5 Fig — Reads for SG_1 library sequenced on the Illumina NextSeq platform were aligned to the VSGnome using bowtie2 [76] and a mapping quality filter of >40 was applied. (TIF) [file ppat.1009904.s005.tif]

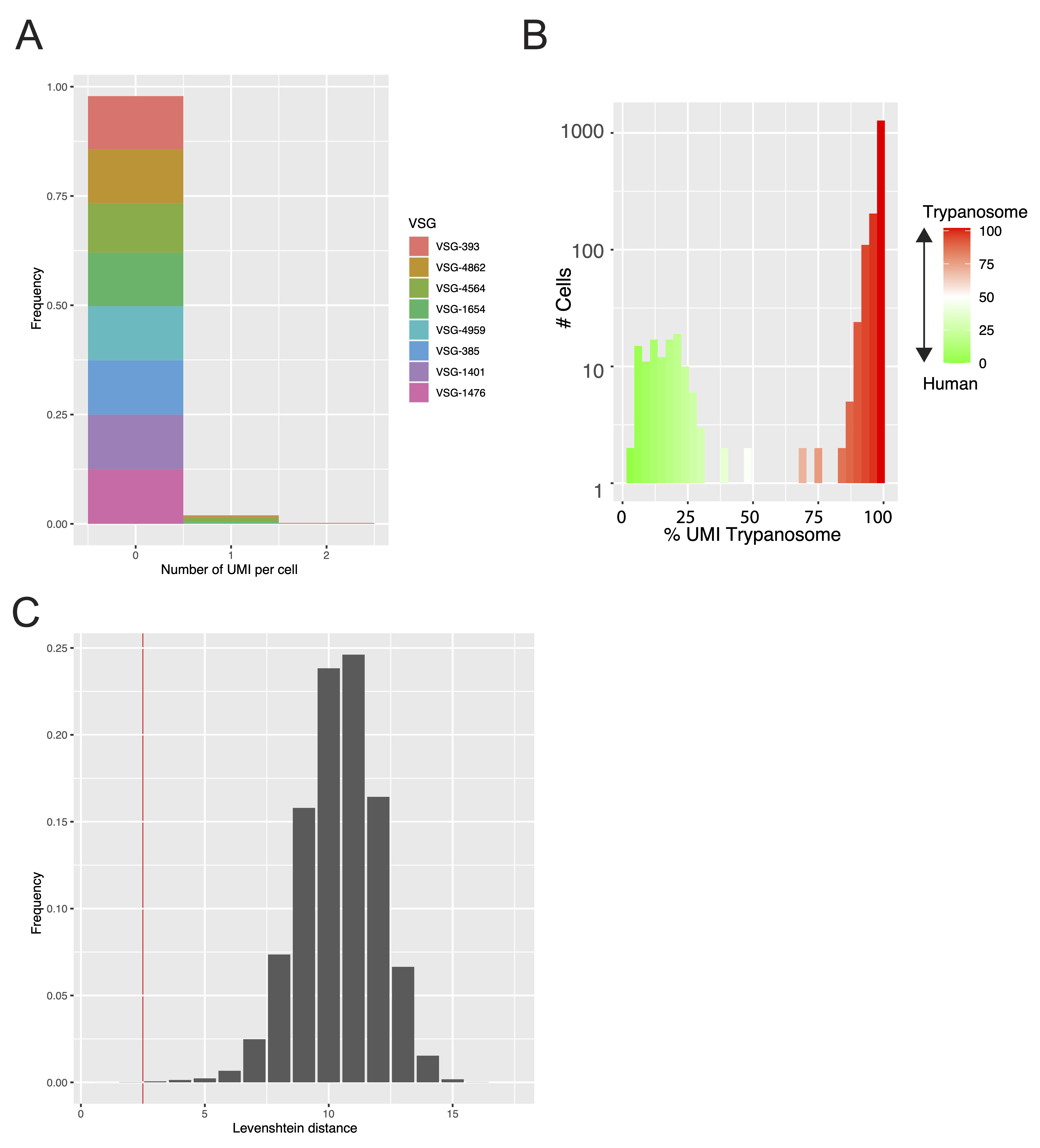

Supplement: S6 Fig — A. Ambient RNA analysis. Plot shows the frequency of VSG UMI detection in a cluster of Ramos cells. B. Histogram shows the percentage of stringently aligned reads (mapQ > 40) to the trypanosome or human transcriptomes. C. Barcode switching analysis. The plot shows the frequency of Levenshtein distances (number of changes required to permute one barcode to another) between all pairs of barcodes in replicate 1 library 1 (1,129 barcodes). Red line shows cut-off for error correction. (TIF) [file ppat.1009904.s006.tif]

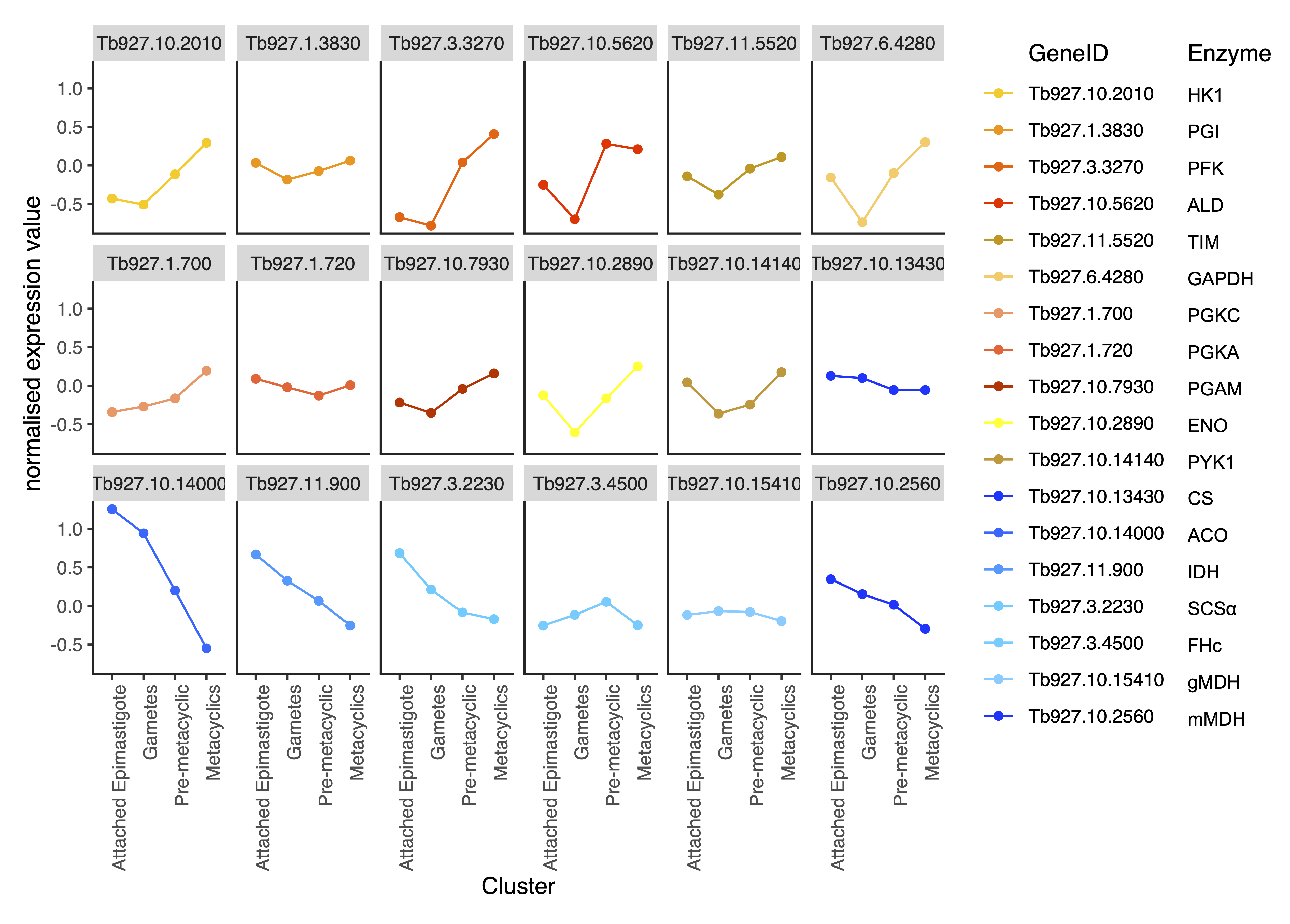

Supplement: S7 Fig — As in Fig 3B except each gene is plotted individually. (TIF) [file ppat.1009904.s007.tif]

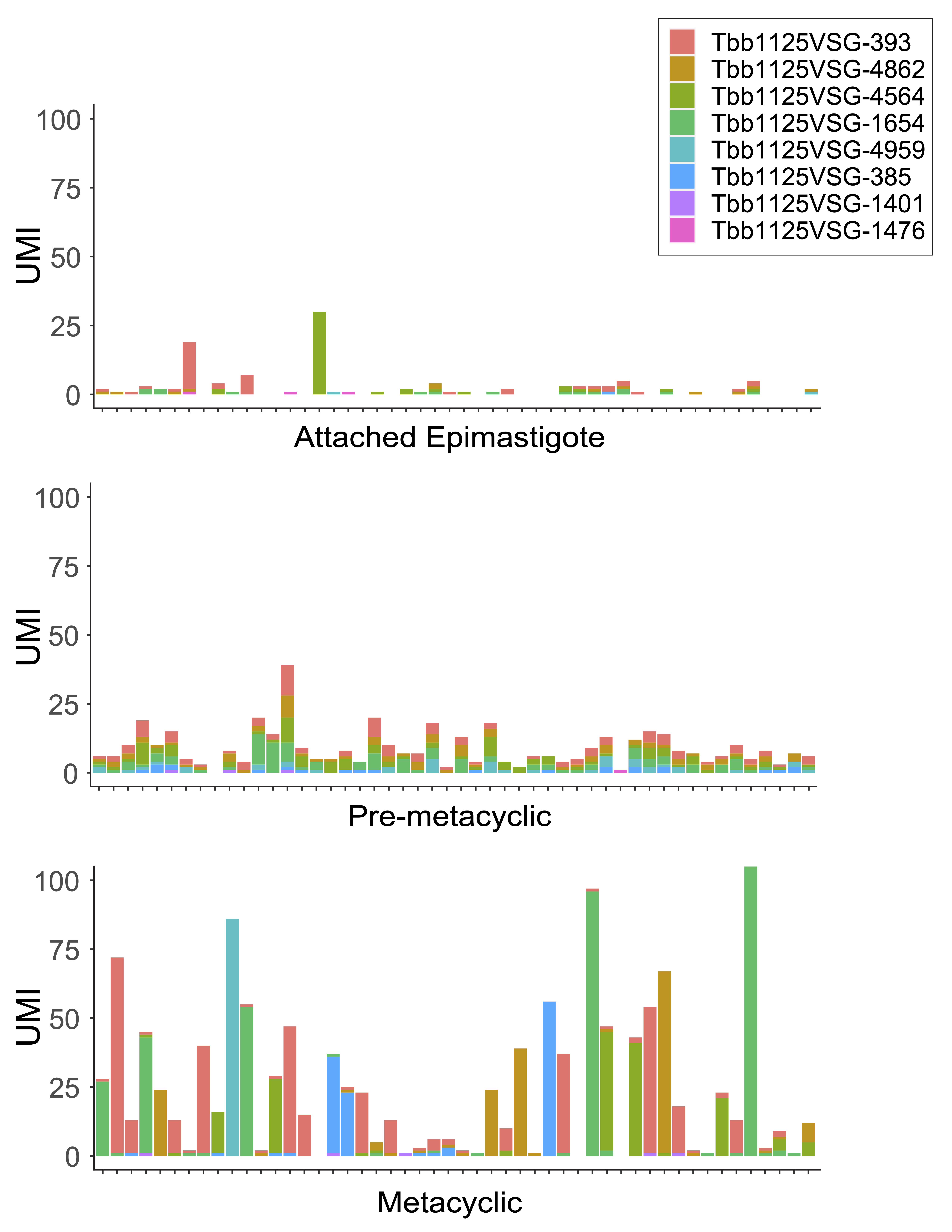

Supplement: S8 Fig — Data presented have not been filtered (no minimum UMI cutoff per cell) and consist of 50 random cells. Units are raw (non-normalised and unscaled) UMI counts per cell. All VSG counts use a stringent mapping quality (MapQ40). (TIF) [file ppat.1009904.s008.tif]

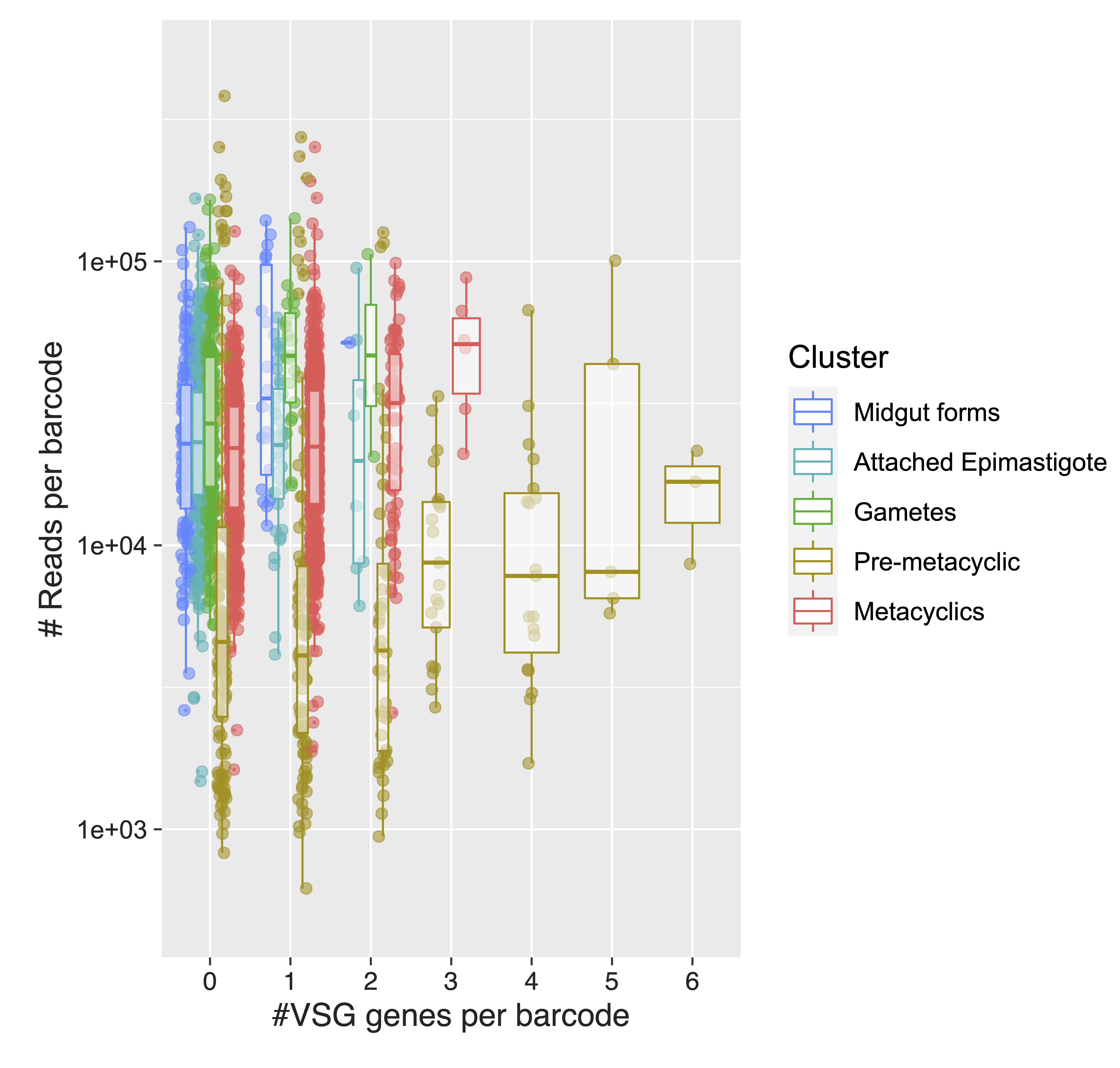

Supplement: S9 Fig — Boxplots show bounds are 25th 50th and 75th percentiles. Individual barcodes are plotted as dots. (TIF) [file ppat.1009904.s009.tif]

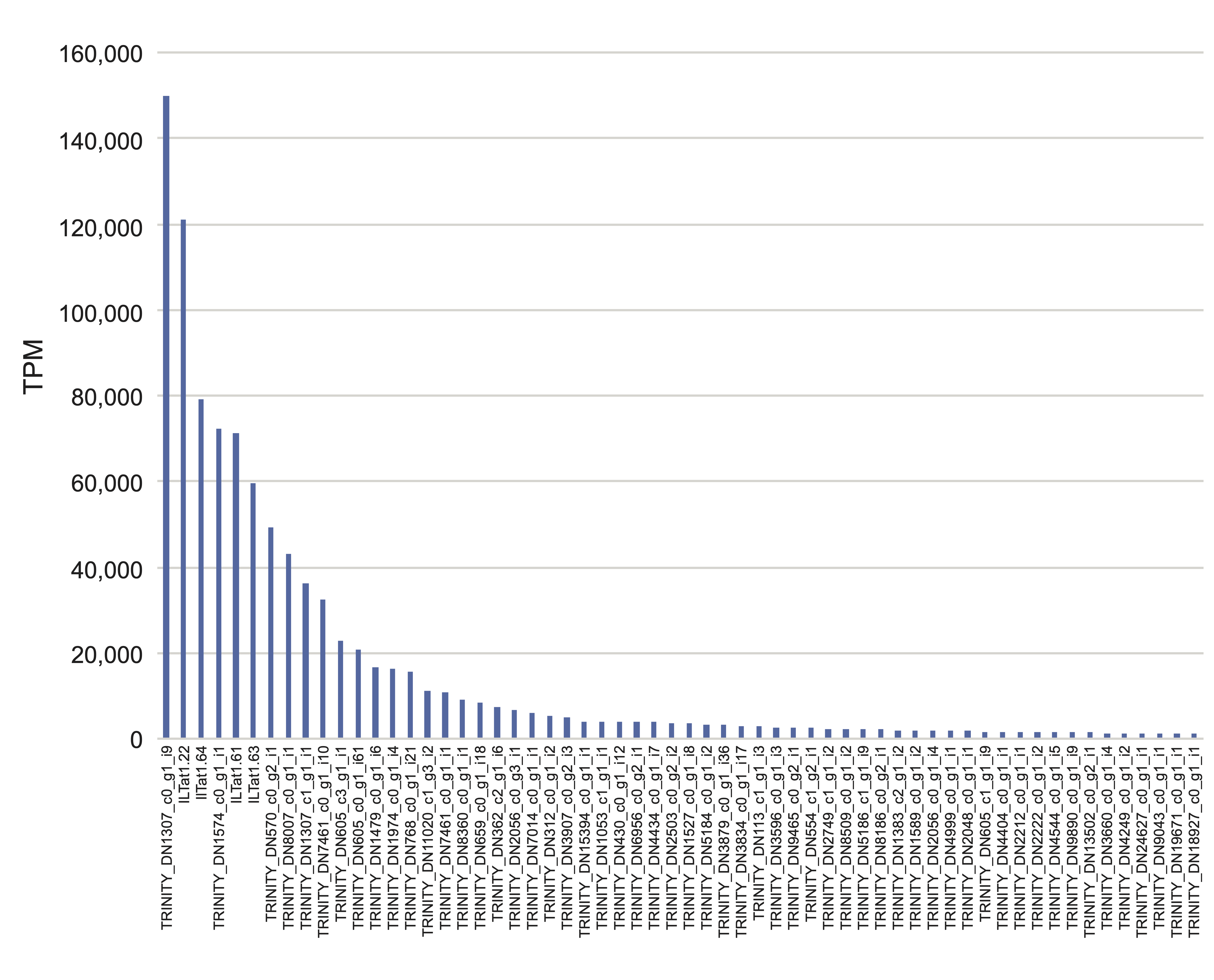

Supplement: S10 Fig — Transcripts were assembled using trinity with bulk RNA-seq data [13] and abundance estimates (TPM) were generated using Kallisto [86]. (TIF) [file ppat.1009904.s010.tif]

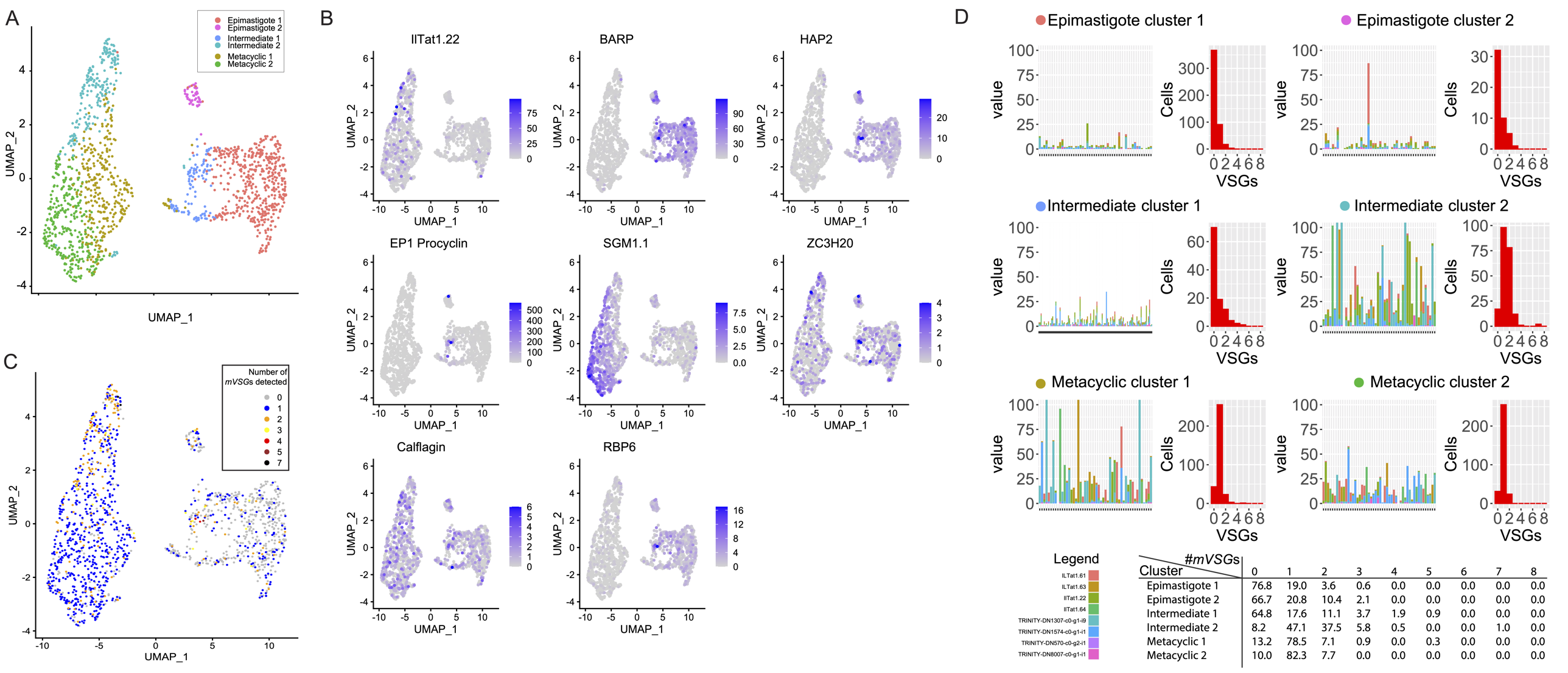

Supplement: S11 Fig — UMAP projection of scRNA-seq data indicating the classifications of clusters. B. Normalised expression values overlaid on UMAP projections for VSG (IlTat1.22 AJ012198.3), BARP (Tb927.9.15640), HAP2 (Tb927.10.10770), EP1-Procyclin (Tb927.10.10260), SGM1.1 (Tb927.7.6490), ZC3H20 (Tb927.7.2660), Calflagin (Tb927.8.5470), and RBP6 (Tb927.3.2930). C. UMAP projection of scRNA-seq data overlaid with the number of mVSG transcripts detected per barcode. D. VSG expression profiles for each cluster. Per cluster: Left, VSG expression data for a random subset of 50 cells. Data are raw (unscaled) UMI counts. Each column represents a cell and each colour a different VSG. Right, histogram shows the number of VSG expressed for all cells (per VSG UMI count > 2, all cells in cluster). Below: table shows the percentages of cells per cluster represented on corresponding histograms. (TIF) [file ppat.1009904.s011.tif]
