# Supplementary material for: The establishment of variant surface glycoprotein monoallelic expression revealed by single-cell RNA-seq of Trypanosoma brucei in the tsetse fly salivary glands
Source: PLoS Pathog. 2021 Sep 20;17(9):e1009904. doi: 10.1371/journal.ppat.1009904 (PMC8509897; doi:10.1371/journal.ppat.1009904)
Supplement: Attachment — Submitted filename: Rebuttal letter 130821.docx [file ppat.1009904.s017.docx]

Dear editors,

We thank the reviewers for their positive consideration of our manuscript and their useful suggestions, our point by point responses are below in red. Modified text in the manuscript file is highlighted.

*Reviewer Comments (if any, and for reference):*

*Reviewer's Responses to Questions*

***Part I - Summary*** *Please use this section to discuss strengths/weaknesses of study, novelty/significance, general execution and scholarship.*

*Reviewer #1: Trypanosoma brucei has a complex life cycle, alternating between two hosts. Most of the published data are derived from bloodstream and procyclic trypanosomes, due to technical challenges of working with other fly stages.
Only recently has some light been shed on the later stages of the parasite development in the tsetse fly, but we are still far from having a clear understanding of this part of the life cycle.
Hutchinson et al. address this gap in the knowledge by applying single cell sequencing to parasites isolated from the salivary glands of the flies. Single cell sequencing is a powerful tool to dissect dynamics that so far have been only described at a population level. Overall, I think the paper is well written and the work is well designed and done.
The authors employ the right controls. They first validate the technique on well-studied trypanosome stages (bloodstream and procyclic forms), and the additional use of Ramos cells as positive control further supports their findings.*

*Reviewer #2: This work revisits the gene expression profile of the different development stages of T. brucei found in infected tsetse, although it focuses on the analysis of salivary gland forms. The paper is well written, the work carefully done and includes the description of a new scRNA seq methodology which has been well adapted for trypanosomes. The analyses mainly concentrates in understanding the monoallelic expression of VSG genes during trypanosome metacyclogenesis in vivo, but also touches on the changes in the metabolic pathways for energy production, also upon differentiation.

Single cell transcriptomics of trypanosome forms isolated from infected flies is not novel (Vigneron A. et al 2020 PNAS). However, in this manuscript, the authors have exploited this methodology to better understand aspects of trypanosome biology that were overlook in the Vigneron work; in fact, it makes a better (and more complete) job when looking into the regulation of the expression of mVSG genes from pre-metacyclics into metacyclics, which was then validated in vitro by FISH using RBP6 over-expressor cells. Therefore, although the work is not mechanistic and descriptive in nature, I find it significant as it increases our knowledge on the regulation of expression of T. brucei mVSGs, an information that could be also relevant for other African trypanosome species that undergo metacyclogenesis in tsetse flies. In addition, it is a good methods paper that could be expanded to other trypanosome forms or kinetoplastid parasites.*

***Part II – Major Issues: Key Experiments Required for Acceptance*** *Please use this section to detail the key new experiments or modifications of existing experiments that should be absolutely required to validate study conclusions.

Generally, there should be no more than 3 such required experiments or major modifications for a "Major Revision" recommendation. If more than 3 experiments are necessary to validate the study conclusions, then you are encouraged to recommend "Reject".*

*Reviewer #1: The main concern is that this approach has already been used last year by Vigneron et al., who successfully sequenced the transcriptome of trypanosomes isolated from the salivary glands at the single cell level. However, I believe that the work of Hutchinson and colleagues brings some interesting additions to the previously published data.

In particular, the authors attention is focused on the expression of metacyclic VSGs before the parasites are transmitted to the mammalian host. The establishment of monoallelic expression in variant multigene families is still poorly understood in several organisms.

This work demonstrates for the first time the presence of an intermediate state in which trypanosomes express several mVSGs at low level before committing to the expression of one single VSG. The previous publication was missing this because of the lack of sequenced genome for the strain used in the experiments.
The authors do multiple validations of their observation, by reanalyzing the previously published data with newly assembled VSGs and by smFISH. From my point of view, the use of biological replicates (instead of technical) would have improved the confidence in the results, confirming that this observation is consistent across samples and is reproducible.
Nevertheless, this finding is very interesting and, in my opinion, it brings enough novelty to justify publication, despite the already available single cell data.*

We in fact present three biological replicates based on three orthogonal lines of evidence: (1) inDrop, (2) 10X genomics scRNA-seq, and (3) single molecule RNA-FISH. This was carried out, using three independent trypanosome strains and therefore provides strong evidence supporting the observed *mVSG* expression pattern during metacyclogenesis. *Another interesting new observation compared to the previously published work is the identification of a gamete cluster. Why do the authors think this cluster has not been identified in the previous work? Have they tried to re-analyze those data in order to check for HAP2 or other gametocytogenesis markers expression? The discussion part should include a comment on this aspect. Especially in this instance, employing a biological replicate would help the impact of the findings.*

We believe that the reasons that the gamete cell cluster was not observed in Vigneron *et al* are likely multi-factorial.

1. We utilized an extended transcriptome where we extended the 3´ UTR sequence of each gene to the 5´ end of the next gene. In the case of *HAP2* (Tb927.10.10770), there is no annotated 3´ polyadenylation signal. This is visible as a grey area in the screenshot of our inDrop reads on the *HAP2* locus shown below. In future studies of HAP2, it will be important to use our data to reannotate the polyadenylation signal to capture more transcripts when counting *HAP2* reads.

We note that we used v7.0 of the genome annotation, which contains Tb927.10.10760, which has subsequently been removed from later annotations. (Vigneron *et al* used release 40).

2. In our experience, fly infections are highly variable, with factors such as duration of infection and time since last feeding affecting the distribution of cell types observed. In single cell studies, this could affect the ability to form reliable clusters, especially if the gamete cell population is relatively small as compared to the rest of the population with abundant amounts of metacyclic or epimastigote trypanosomes.

We have added these points to our discussion, lines 484-490.

*Reviewer #2: 1) It is intriguing that the cluster of "gamete" cells shows an increase in the expression of EP1 over BARP transcripts. Given that there are no known surface markers for "gamete" cells, it would be interesting to stain these cells with anti-EP procyclin antibodies and also anti-BARP, using the BARP staining of epis as positive control.*

We agree that this is indeed an interesting observation, however, we believe a more detailed interrogation of the biology of gamete cells is beyond the scope of this manuscript. Interestingly, we note that EP1 (Tb927.10.10260) procyclin transcripts have been previously observed in trypanosomes from the salivary gland (Urwyler *et al* 2007 Mol. Microbiology), and immunofluorescence analysis of parasites from the salivary gland revealed low-level expression of EP procyclin (Urwyler *et al* 2005 PLoS Pathogens).

We have added these points to the discussion, lines 480-484.

*2) Although I'm not requesting this, I wonder if the authors tried to identify what VSG proteins are expressed by metacyclics at 28 dpi using either proteomics or IF with antibodies specific for the main VSG isoforms detected by scRNA seq. If available, this will better the paper and further complement the VSG expression analysis, but at the protein level.*

Our main goal was to understand the dynamics of *mVSG* gene transcription during metacyclogenesis, we therefore did not assess protein expression in these cells.

***Part III – Minor Issues: Editorial and Data Presentation Modifications*** *Please use this section for editorial suggestions as well as relatively minor modifications of existing data that would enhance clarity.*

*Reviewer #1: (No Response)*

*Reviewer #2: .Introduction: the section describing the route trypanosomes take in the tsetse gut is incorrect (ln 53-55). Parasites colonise the "anterior" not the "posterior" midgut. Also, procyclics use the "ectoperitrophic space" to migrate from the midgut to the PV not the "alimentary tract". Last, formation of epimastigotes only happens within a specific window of time in the PV; i.e. most of cells in the PV don't differentiate into epis. Please amend all these accordingly.*

We have amended the relevant text in the introduction, lines 71-75

*.Please specify which of the EP1 isoforms (EP1-1 or EP1-2) is highly expressed in salivary gland forms.*

As EP1 isoforms are heterozygous alleles of the same locus, and the *T. brucei* reference genome used here is a haploid mosaic, we can only report on the EP1 sequence available. According to Acosta-Serrano (1999) JBC, the EP1-1 isoform has 30 EP repeats and EP1-2 has 25, while the EP1 gene in the reference genome has 28. Additionally, although not impossible, it is unlikely that sufficient numbers of short Illumina reads targeting the 3´end of transcripts would span the EP repeats to be able to confidently distinguish between isoforms.

*.Discussion:
The relevance of the metabolic switching during pre- to metacyclics is poorly discussed. e.g. implications for the type of carbon source used for ATP production and pre-adaptation for transmission to the vertebrate host?*

While we wished to report our findings to the community, the quality of evidence for the timing of metabolic remodeling during metacyclogenesis relies only on transcript level (and not on direct protein evidence). Therefore, we restricted the extent of our discussion. We feel that the recent study focused on metabolism adaptation during fly infection (Doleželová et al., PloS Biol. 2020) was better placed to develop these kinds of ideas.

*Although a hypothesis, the suggestion that VEX1 and VEX2 control monoallelic expression of VSG in metacyclics is highly speculative. Is there any evidence that these proteins are indeed expressed in pre- or metacyclic forms?*

While we agree that this is a speculative hypothesis, we also think it is the most parsimonious way to explain all available data. To directly answer the reviewers’ question, there is peptide evidence of these proteins in metacyclic forms (Doleželová *et al* 2020).

In addition, it is clear that the control of monoallelic *VSG* gene expression is a complex system that operates in multiple life cycle stages of the parasite. We consider it unlikely that a second system would have evolved to control monogenic gene expression in the metacyclic forms which is then switched off for a VEX based system in BSF.
